# The unstructured C-terminal extension of UvrD interacts with UvrB, but is dispensable for nucleotide excision repair

**DOI:** 10.1016/j.dnarep.2009.08.005

**Published:** 2009-11-02

**Authors:** Laura Manelyte, Colin P. Guy, Rachel M. Smith, Mark S. Dillingham, Peter McGlynn, Nigel J. Savery

**Affiliations:** a DNA-protein Interactions Unit, Department of Biochemistry, University of Bristol, Bristol BS8 1TD, UK; b Institute of Medical Sciences, University of Aberdeen, Foresterhill, Aberdeen AB25 2ZD, UK

**Keywords:** Nucleotide excision repair, Helicase recruitment, Protein–protein interactions

## Abstract

During nucleotide excision repair (NER) in bacteria the UvrC nuclease and the short oligonucleotide that contains the DNA lesion are removed from the post-incision complex by UvrD, a superfamily 1A helicase. Helicases are frequently regulated by interactions with partner proteins, and immunoprecipitation experiments have previously indicated that UvrD interacts with UvrB, a component of the post-incision complex. We examined this interaction using 2-hybrid analysis and surface plasmon resonance spectroscopy, and found that the N-terminal domain and the unstructured region at the C-terminus of UvrD interact with UvrB. We analysed the properties of a truncated UvrD protein that lacked the unstructured C-terminal region and found that it showed a diminished affinity for single-stranded DNA, but retained the ability to displace both UvrC and the lesion-containing oligonucleotide from a post-incision nucleotide excision repair complex. The interaction of the C-terminal region of UvrD with UvrB is therefore not an essential feature of the mechanism by which UvrD disassembles the post-incision complex during NER. In further experiments we showed that PcrA helicase from *Bacillus stearothermophilus* can also displace UvrC and the excised oligonucleotide from a post-incision NER complex, which supports the idea that PcrA performs a UvrD-like function during NER in Gram-positive organisms.

## 1 Introduction

UvrD (helicase II) is a superfamily 1A helicase that unwinds DNA during nucleotide excision repair (NER) and mismatch repair (MMR) in Gram-negative bacteria. During NER (reviewed in [1]) the DNA lesion is detected by a complex of UvrA and UvrB, and then UvrA dissociates, allowing UvrC to bind to the UvrB:DNA complex. UvrC nicks the damaged strand twice: first at the 4th or 5th phosphodiester bond 3′ of the lesion and then at the 8th phosphodiester bond 5′ of the lesion. UvrD is recruited to the UvrB:UvrC:DNA post-incision complex and displaces both UvrC and the 12–13 nt oligonucleotide containing the damaged base(s). A repair patch is then generated by DNA polymerase I and DNA ligase. During MMR (reviewed in [2]) the mismatched base pair is detected by MutS, which then recruits MutL. The MutS:MutL complex communicates with MutH bound at the nearest hemimethylated GATC sequence, causing it to generate a nick in the backbone of the unmethylated (newly synthesised) DNA strand. UvrD is recruited to this nick and unwinds the DNA between the nick and the mismatched base pair. The unmethylated strand that is displaced by UvrD is degraded, and a repair patch is generated by DNA polymerase III and DNA ligase.

In both the NER and MMR pathways the recruitment and regulation of UvrD is mediated by interaction with other proteins. This is best characterised in MMR, where MutL loads UvrD onto the nicked DNA and stimulates its DNA-unwinding activity (reviewed in [3]). The interaction between the two proteins has been demonstrated in 2-hybrid and biochemical assays, and progress has been made towards identifying the regions of MutL that contact UvrD [4]. The interactions made by UvrD during NER are less well characterised: a combination of UvrA and UvrB stimulates UvrD helicase activity on a range of DNA substrates containing strand discontinuities [5], and immunoprecipitation experiments using His-tagged UvrD suggest that UvrD can bind to both UvrA and UvrB, but not to a UvrA:UvrB complex [6].

In this work we have used 2-hybrid analysis, surface plasmon resonance spectroscopy and functional assays to investigate the interaction between the UvrD and UvrB proteins of *E. coli*. We show that UvrD interacts with the N-terminal domains of UvrB, and this interaction involves both the N-terminal domain of UvrD and the unstructured C-terminal extension of UvrD. A truncated form of UvrD that lacks the C-terminal extension shows reduced affinity for single-stranded DNA, but retains helicase activity on a variety of substrates. The truncated UvrD derivative was able to displace both UvrC and the damage-containing oligonucleotide from the UvrB:UvrC:DNA post-incision complex, indicating that the interaction between UvrB and the C-terminal region of UvrD is dispensable for these activities.

## 2 Materials and methods

### 2.1 Plasmids

The *uvrA*, *uvrB* and *uvrC* coding regions were amplified from *E. coli* MG1655 genomic DNA using PCR with primers that introduced a BamHI site immediately upstream of the start codon and a HindIII site immediately downstream of the stop codon. The upstream primer used for amplification of *uvrC* also mutated the natural GTG start codon to ATG. The PCR fragments and plasmid pQE30 (Qiagen) were digested with BamHI and HindIII, and ligated to create plasmids pQE30UvrA, pQE30UvrB and pQE30UvrC. These encode N-terminally hexa-His-tagged UvrA, UvrB and UvrC, respectively, under the control of an IPTG-inducible T5 promoter. Plasmid pQE30UvrB_1–414_ encodes a truncated N-terminally His-tagged UvrB protein consisting of residues 1–414. It was derived from pQE30UvrB by the “rolling circle PCR” method [7] using primers that delete codons 415–673 and place a stop codon and HindIII site immediately downstream of codon 414.

The *uvrD* gene was amplified from *E. coli* MG1655 genomic DNA using PCR with primers that introduced an NcoI site overlapping the start codon and an XhoI site immediately downstream of the stop codon. The PCR product was digested with NcoI and XhoI and ligated into the NcoI/XhoI sites of pETDUET or pET28a to create pETDUET-UvrD and pET28a-UvrD, respectively. Both constructs encode native (untagged) UvrD under the control of an IPTG-inducible T7 promoter. Plasmid pETDUET-UvrD_1–647_ encodes a truncated untagged UvrD protein consisting of residues 1–647. It was derived from pETDUET-UvrD by the “rolling circle PCR” method [7] using primers that delete codons 648–720 and place a stop codon and XhoI site immediately downstream of codon 647.

Expression constructs for producing full-length and C-terminally truncated UvrD biotinylated at the N-terminus were created by modifying pET28a-UvrD and pETDUET-UvrD_1–647_. The parent vectors were cut with NcoI and a short insert composed of annealed oligonucleotides was ligated into that position to create pET28a-^bio^UvrD and pETDUET-^bio^UvrD_1–647_. The insert introduced a 20 amino acid tag (MSG LND IFE AQ**K** IEW HEG GG) at the N-terminus of UvrD. The lysine residue within this tag is biotinylated *in vivo* by the *E. coli* BirA enzyme [8].

pRA02 is a pBR322-based (Ap^R^) construct that encodes residues 1–248 of the RNA polymerase α subunit under the control of an *lppP-lacUV5* tandem promoter. A linker at the 3′ end of the *rpoA* coding region encodes an alanine residue followed by an XbaI site and a KpnI site that allow proteins of interest to be cloned in frame with the truncated *rpoA* gene. The plasmid was constructed in several steps, as follows. The XbaI site in the 5′ untranslated region of the *rpoA* gene in pREIIα [9] was destroyed by digestion with XbaI, treatment with Klenow polymerase plus dNTPs, and religation. A fragment of the modified plasmid encompassing the *lppP-lacUV5* tandem promoter and the coding region for residues 1–248 of the α subunit was amplified by PCR using primers that introduced an alanine codon downstream of codon 248, followed by XbaI and KpnI sites. Plasmid pSRlacUV5_(−140/63)_ [10] was digested with EcoRI and HindIII and treated with Klenow polymerase plus dNTPs to remove the lacUV5 promoter and generate blunt ends. The *rpoA*-containing PCR product was ligated with the pSR backbone to generate pRA02.

pRA03 is a pACYC184-based (Cm^R^) construct that encodes residues 1–236 of λ cI under the control of the *lacUV5* promoter. A linker at the 3′ end of the λ cI coding region encodes 3 alanine residues followed by an XbaI site and a KpnI site that allow proteins of interest to be cloned in frame with the truncated λ cI gene. The plasmid was constructed in several steps, as follows. Plasmid pBRcI-ω [11] was digested with NotI and SalI and was ligated with a double-stranded oligonucleotide that incorporated an XbaI site and a KpnI site. The modified plasmid was digested with EcoRI, and plasmid pACYC184 was digested with HindIII. Each plasmid was then treated with Klenow polymerase plus dNTPs to generate blunt ends, and subsequently digested with SalI. The blunt/SalI fragment carrying the *lacUV5* promoter, truncated λ cI gene and XbaI/KpnI linker was ligated with the blunt/SalI pACYC184 backbone to generate pRA03.

Derivatives of pRA02 that encode α-UvrD fusion proteins and derivatives of pRA03 that encode λ cI-UvrB fusion proteins were created by PCR amplification of the indicated regions of the *uvrD* or *uvrB* genes using primers that introduced an XbaI site at the 5′ end of the region and a KpnI site at the 3′ end. The XbaI/KpnI-digested products were then inserted into the XbaI/KpnI sites of pRA02 or pRA03.

The coding sequences of all plasmids constructed in this work were confirmed by sequencing (The Sequencing Service, University of Dundee). The sequences of the primers used in vector construction are available on request.

### 2.2 Bacterial 2-hybrid assay

The bacterial 2-hybrid assay used in this work is essentially that developed by Dove and Hochschild [11,12]. Reporter strain KS1 [12] carries a chromosomal *lacZ* gene under the control of a promoter that can be activated by interactions between a protein fused to λ cI and a protein fused to the α subunit of RNA polymerase. KS1 cells were transformed with the indicated combinations of pRA02 and pRA03 derivatives. An overnight culture was used to inoculate 5 ml of LB broth supplemented with IPTG (1 mM), ampicillin (100 μg/ml), chloramphenicol (25 μg/ml) and kanamycin (50 μg/ml) and the cultures were incubated at 30 °C to mid-log phase (A_600_ ∼0.3). β-galactosidase activity was assayed as described by Miller [13].

### 2.3 Proteins

N-terminally His-tagged UvrA, UvrB, UvrB_1–414_ and UvrC proteins were purified from XL1-Blue cells transformed with pQE30UvrA, pQE30UvrB, pQE30UvrB_1–414_ and pQE30UvrC, respectively. A single colony was used to inoculate 100 ml of LB broth supplemented with 100 μg/ml ampicillin and 25 μg/ml tetracycline and the cultures were incubated at 37 °C for 16 h. The cells were harvested by centrifugation at 4 °C, and the pellet was resuspended in 20 ml of wash buffer (20 mM Tris–HCl pH 8.0, 300 mM NaCl, 20 mM imidazole) containing 10 mg of lysozyme and incubated on ice for 30 min. The cells were lysed on ice by sonication and the soluble fraction was recovered by centrifugation. The His-tagged protein was purified from the lysate using a 1 ml, NiSO_4_ charged, HiTrap chelating column (Amersham) on an ÄKTA FPLC (Amersham). The protein was eluted from the column using a 20–500 mM imidazole gradient in wash buffer. Fractions containing only the protein of interest were dialysed at 4 °C overnight against storage buffer (10 mM Tris–HCl pH 8.0, 1 mM EDTA, 200 mM KCl, 50% (v/v) glycerol, 2 mM DTT).

Untagged UvrD and UvrD_1–647_ were purified from BL21(DE3) cells transformed with pETDUET-UvrD or pETDUET-UvrD_1–647_ vectors. An overnight culture was used to inoculate 1 l of LB broth supplemented with 100 μg/ml ampicillin. The cultures were grown at 37 °C until A_600_ was ∼0.7, induced by adding 1 mM IPTG, and incubated at 37 °C for 1 h. The cells were harvested by centrifugation at 4 °C, and the pellet was resuspended in 20 ml of lysis buffer (50 mM Tris–HCl pH 8.3, 5% (v/v) glycerol, 200 mM NaCl, 5 mM EDTA, 1 mM DTT) containing 10 mg of lysozyme and incubated on ice for 30 min. 250 μl 4% sodium deoxycholate and 20 μl 5 mg/ml DNaseI were added and then incubated on ice for another 30 min. To increase the UvrD solubility, NaCl concentration was increased to ∼450 mM by adding 1.2 ml 5 M NaCl and stirring for 15 min at 4 °C. The cells were lysed on ice by sonication and the soluble fraction was recovered by centrifugation. Supernatant was slowly diluted in buffer A (20 mM Tris–HCl pH 8.3, 1 mM EDTA, 20% (v/v) glycerol and 1 mM DTT) until the salt concentration was approximately 100 mM NaCl and was then loaded onto a Heparin column (Amersham) on an ÄKTA FPLC. The protein was eluted from the column using a 0.1–2 M NaCl gradient in buffer A. Fractions containing only the protein of interest were combined, diluted in buffer A until the salt concentration was approximately 100 mM NaCl and then loaded onto a MonoQ column (Amersham) on an ÄKTA FPLC. The protein was eluted using a 0.1–2 M NaCl gradient in buffer A. Fractions containing only the protein of interest were dialysed at 4 °C overnight against storage buffer.

Biotinylated proteins ^bio^UvrD and ^bio^UvrD_1–647_ were purified from BL21(DE3) cells co-transformed with plasmid pBirACm (Avidity: a pACYC184-derived plasmid that carries an IPTG-inducible biotin ligase gene) and either pET28a-^bio^UvrD or pETDUET-^bio^UvrD_1–647_. All purification steps were performed at 4 °C, except for heparin affinity chromatography which was performed at room temperature. An overnight culture was used to inoculate 2 l of LB broth supplemented with appropriate antibiotics (34 μg/ml chloramphenicol and either 30 μg/ml kanamycin or 100 μg/ml ampicillin). The cultures were grown at 37 °C to mid-log phase, at which point 1 mM IPTG and 50 μM biotin (Sigma) were added. Cells were grown for a further 3 h at 37 °C, and then harvested by centrifugation. In the case of ^bio^UvrD cells the cell pellet was resuspended in 40 ml cell resuspension buffer (50 mM Tris–HCl pH 7.5, 100 mM NaCl, 1 mM EDTA, 1 mM DTT, 10% sucrose) supplemented with a protease inhibitor cocktail according to manufacturer's instructions (Promega). Cells were lysed by sonication and the soluble fraction recovered by centrifugation. Solid ammonium sulphate was added slowly to 50% saturation with stirring. The precipitated protein was recovered by centrifugation and resuspended in buffer C (50 mM Tris–HCl pH 7.5, 1 mM EDTA, 1 mM DTT) + 100 mM NaCl. A Softlink Avidin resin column (∼15 ml, Promega) was poured and prepared for use according to the manufacturer's instructions. The column was equilibrated in buffer C + 100 mM NaCl and the sample loaded. After extensive washing with buffer C + 100 mM NaCl, the ^bio^UvrD was eluted with buffer C + 100 mM NaCl + 5 mM biotin. The peak fractions were loaded directly onto a 5 ml HiTrap heparin column equilibrated with buffer C + 100 mM NaCl. The column was washed extensively with buffer C + 100 mM NaCl to remove free biotin and the ^bio^UvrD was then eluted with a gradient to buffer C + 1 M NaCl. The peak fractions were collected and dialysed overnight against buffer A + 200 mM NaCl + 10% (v/v) glycerol. In the case of ^bio^UvrD_1–647_ the cell pellet was resuspended and lysed as described for untagged UvrD_1–647_. The protein was purified from this lysate using Softlink Avidin Resin and HiTrap heparin columns as described for ^bio^UvrD, except that buffer A was used in place of buffer C and the final protein solution was dialysed against storage buffer.

*B. stearothermophilus* PcrA and *E. coli* Rep were purified essentially as described previously [5,14].

### 2.4 Surface plasmon resonance spectroscopy

Surface plasmon resonance spectroscopy assays were performed using a BIAcore 2000 instrument. Streptavidin (SA) sensor chips were used to immobilize ^bio^UvrD and ^bio^UvrD_1–647_. SPR buffer (10 mM HEPES pH 7.4, 150 mM NaCl, 3 mM EDTA, 0.005% Tween20) was used as the binding buffer. The amount of ligand immobilised was ∼7000 resonance units (RU). The control flow cell was treated the same way as assay flow cells but without protein immobilized. The indicated concentrations of UvrB or UvrB_1–414_ were injected at 20 μl/min in SPR buffer at 25 °C. Data were collected for 1 min association and 2.5 min dissociation. In other assays, approximately 8000 RUs of UvrB or UvrB_1–414_ were immobilized on a CM5 chip by amine coupling at pH 4.5 (UvrB) or 4.0 (UvrB_1–414_) and the indicated concentrations of UvrD or UvrD_1–647_ were injected at 20 μl/min in SPR buffer at 25 °C. To analyze the data, the sensorgrams of the no-protein control flow cell were subtracted from those of the assay flow cells in order to eliminate the effects of non-specific interactions.

### 2.5 Oligonucleotide substrates

Double-stranded oligonucleotide substrates were prepared by mixing single-stranded oligonucleotides in annealing buffer (50 mM NaCl, 10 mM Tris–HCl pH 7.5, 1 mM MgCl_2_), heating at 90 °C for 2 min and then cooling slowly to room temperature. Oligonucleotides that were not fluorescently or radioactively labelled were present in a 1.2-fold molar excess over the labelled oligonucleotide. A double-stranded 50 bp DNA substrate containing fluorescein-dT (FldT) was created by annealing RA003 (5′-CTC ATA CGA CGC TGT CGA TCC AGT CAC TGT CAT GCG CTA TCC GAT CCT AG-3′) and RA004-FldT (5′-CTA GGA TCG GAT AGC GCA TGA CAG TGA C FldT G GAT CGA CAG CGT CGT ATG AG-3′). For DNA-unwinding experiments: the 3′ overhang substrate was generated by annealing [γ-^32^P]-ATP end-labelled RA003rev22 (5′-TGG ATC GAC AGC GTC GTA TGA G-3′) and RA003; the blunt-end substrate was made by annealing [γ-^32^P]-ATP end-labelled RA003rev22 and RA003-22 (5′-CTC ATA CGA CGC TGT CGA TCC A-3′); nicked substrates were made by annealing RA003 with [γ-^32^P]-ATP end-labelled RA003rev22 and RA003rev28 (5′-CTA GGA TCG GAT AGC GCA TGA CAG TGA C-3′), or by annealing RA003 with [γ-^32^P]-ATP end-labelled RA003rev13 (5′-AGC GTC GTA TGA G-3′) and RA003rev37 (5′-CTA GGA TCG GAT AGC GCA TGA CAG TGA CTG GAT CGA C-3′). All oligonucleotides were purchased from MWG-Biotech and were HPLC-purified by the manufacturer.

### 2.6 Assay for oligonucleotide displacement from the post-incision complex

To generate post-incision NER complexes 10 nM freshly annealed FldT-containing 50mer was incubated with 40 nM UvrA, 200 nM UvrB and 100 nM UvrC for 1 h at 37 °C in 40 mM HEPES pH 8.0, 100 mM KCl, 8 mM MgCl_2_, 5 mM DTT, 100 μg/ml BSA, 11% (v/v) glycerol and 2 mM ATP. The reaction also contained an ATP-regeneration system (0.022 units/μl pyruvate kinase (Sigma), 0.029 units/μl lactate dehydrogenase (Sigma), 1 mM phosphoenolpyruvate). UvrD, UvrD_1–647_, PcrA or Rep (10 nM final concentration) were added to aliquots of post-incision complex mix, and the reactions were incubated at 37 °C. At the indicated intervals aliquots were removed and loaded onto non-denaturing 10% polyacrylamide gels containing 0.5× TBE and 10 mM MgCl_2_. To monitor the proportion of DNA cleaved in the post-incision complex additional aliquots were mixed with an equal volume of 95% formamide/20 mM EDTA, incubated at 95 °C for 5 min and loaded on denaturing 10% polyacrylamide gels containing 1× TBE and 7 M urea. Wet gels were scanned (Molecular Dynamics Typhoon) at 720 V using a fluorescein emission filter and either blue1 (488 nm) or green (532 nm) excitation lasers for non-denaturing and denaturing gels, respectively. Gel images were analyzed using ImageQuant software.

### 2.7 Helicase assays

Helicase activity was monitored under two sets of conditions. The experiments shown in Fig. 4 were conducted under the “high-salt” conditions used to monitor displacement of the FldT-containing oligonucleotide from a post-incision complex. In these experiments 10 nM γ^32^P-labelled nicked substrate was pre-incubated at 37 °C in 40 mM HEPES pH 8.0, 100 mM KCl, 8 mM MgCl_2_, 5 mM DTT, 100 μg/ml BSA, 11% (v/v) glycerol and 2 mM ATP containing the ATP-regeneration system described above. The reaction was started by the addition of helicase (10 nM final) and aliquots were removed at the stated intervals and terminated by the addition of an equal volume of stop buffer (400 mM EDTA pH 8.0, 10% (v/v) glycerol, 200 nM oligonucleotide RA003rev22). The experiments shown in Fig. 6 were conducted under “low-salt” conditions. In these experiments the indicated concentrations of UvrD or UvrD_1–647_ were pre-incubated with 2 nM γ^32^P-labelled DNA substrate for 5 min at 37 °C in 25 mM Tris–HCl pH 7.5, 50 mM KCl, 4 mM MgCl_2_, 2 mM DTT, 5% (v/v) glycerol, 100 μg/ml BSA. The reaction was started by the addition of ATP (2 mM final) and reactions were incubated at 37 °C for 10 min before being terminated by the addition of an equal volume of stop buffer. In all experiments the terminated reactions were analysed on native 20% polyacrylamide gels containing 1× TBE. Gels were dried and analyzed using Molecular Dynamics Typhoon PhosphorImager and ImageQuant software.

### 2.8 ATPase assay

ATPase activity was measured using an ATP-NADH-coupled assay. The assays were carried out at 37 °C in a 200-μl reaction volume containing 1 nM wild-type UvrD or UvrD_1–647_ in 25 mM Tris–HCl pH 8.0, 50 mM KCl, 4 mM MgCl_2_, 4% (v/v) glycerol, 2 mM DTT, 100 μg/ml BSA) with 4.4 units pyruvate kinase and 5.7 units lactate dehydrogenase (Sigma), 500 μM phosphoenolpyruvate, 400 μM NADH (Sigma) and poly(dT) (Sigma) at 0, 0.05, 0.1, 0.25, 0.5, 1, 2, 4, 8 and 16 μM nucleotides. The reactions were started by the addition of ATP at a final concentration of 2 mM and the A_340_ was monitored at 15 s intervals for 1 h in a VERSAmax 96-well plate reader (Molecular Devices). The accompanying software (SOFTmaxPro) was used to obtain linear fits and the rate of ATP hydrolysis was calculated from the change of absorbance.

### 2.9 UvrC-turnover assay

A DNA template containing randomly located UV-induced photoproducts was generated by irradiating a 4.2 kb ^3^H-labelled [15] plasmid in 20 μl 17 nM aliquots with 30 J m^−2^ 254 nm UV light. The UV-irradiated plasmid DNA (1.2 nM) was mixed with 6 nM UvrA, 30 nM UvrB, 3 nM UvrC and the indicated concentrations of UvrD, UvrD_1–647_, PcrA and Rep in repair buffer (40 mM HEPES pH 8.0, 50 mM KCl, 8 mM MgCl_2_, 5 mM DTT, 100 μg/ml BSA, 4% v/v glycerol). The reactions were started by the addition of ATP at a final concentration of 2 mM and incubated for 10 min at 37 °C. The reactions were stopped by the addition of 0.25× volume of 5× STEB (100 mM Tris–HCl pH 8.0, 100 mM EDTA, 1.17 M sucrose, 0.4 mg/ml bromophenol blue). Samples were analysed on 1% agarose gels in 1× TAE and the bands corresponding to supercoiled and nicked DNA in each sample were excised from the gel and quantified by scintillation counting.

### 2.10 UV sensitivity assay

LB broth (5 ml) containing 100 μg/ml ampicillin and 50 μg/ml kanamycin was inoculated with JW3786-5 (*ΔuvrD769∷kan*) [16] cells transformed with pETDUET-UvrD, pETDUET-UvrD_1–647_ or pETDUET. Control cultures of the *uvrD*^*+*^ parent strain BW25113 [17] transformed with pETDUET were grown in LB broth (5 ml) containing 100 μg/ml ampicillin. The cells were incubated at 30 °C for 16–18 h and then 50 μl transferred into 10 ml LB broth containing the appropriate antibiotics. The cells were incubated at 37 °C to an A_600_ of ∼0.5, and then placed on ice. An aliquot of each culture corresponding to (0.5/measured A_600_) ml was centrifuged at 16,110 × *g* for 1 min and the cell pellet was resuspended in 1 ml M9 broth. The cell suspensions were serially diluted in M9 broth in 10-fold steps. 2 μl of each dilution was spotted onto replicate LB agar plates containing 100 μg/ml ampicillin. Plates were irradiated with the stated doses of 254 nm UV light using a Stratalinker (Stratagene). The plates were incubated at 30 °C for 16 h and UV survival was calculated by counting the number of colonies within the most diluted viable spot. The results presented are the average of at least 3 independent experiments, each conducted in triplicate.

## 3 Results

### 3.1 UvrD interaction with UvrB involves the C-terminal region of UvrD

The interaction of UvrD with UvrB has been demonstrated in immunoprecipitation experiments using full-length purified proteins [6]. In order to determine which regions of these proteins interact with one another we used a bacterial 2-hybrid assay that detects interactions between one protein fused to the λcI repressor protein and a second fused to the N-terminal domain of the RNA polymerase α subunit [11,12]. During this assay plasmids encoding the two fusion proteins are introduced into a reporter strain containing a chromosomal *lacZ* gene under the control of an artificial promoter containing a binding site for the λcI protein upstream of the core promoter elements. Any interaction between the two fusion proteins results in RNA polymerase being recruited to the promoter, and can therefore be detected by monitoring *lacZ* expression.

**Fig. 1:**
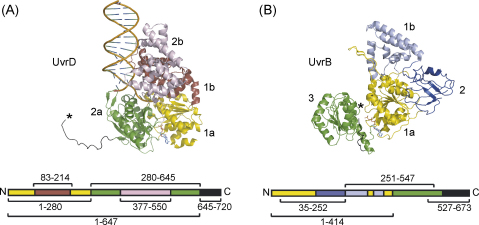
Structural organisation of UvrD and UvrB, showing the truncated proteins used in this work. (A) Structure of a UvrD:DNA complex (from pdb 1IS6 [18]). Domains 1a (yellow) and 2a (green) form the nucleotide-binding site (occupied by ADP.MgF_3_ in the structure shown), and are interrupted by domains 1b (red) and 2b (magenta), respectively. The structure shown lacks the C-terminal 58 residues: the C-terminus is indicated by an asterisk. (B) Structure of UvrB (from pdb 1D9Z [19]). Domains 1a (yellow) and 3 (green) form the nucleotide-binding site (occupied by ATP in the structure shown). Domains 1b and 2 are shown in light blue and dark blue, respectively. The structure shown lacks the C-terminal domain (domain 4): the C-terminus is indicated by an asterisk. (For interpretation of the references to color in this figure legend, the reader is referred to the web version of the article.)

UvrD consists of 4 domains and a C-terminal extension (Fig. 1A) [18]. Two RecA-like domains (1a and 2a) comprise the ATPase module of the protein, and each of these contains an insertion that folds into a separate domain (1b and 2b). At the C-terminus of the protein is a region of approximately 70 residues for which no structure has been determined (the C-terminal 40 residues were not present in the construct used for crystallisation, and the remainder either formed random coils or were not resolved in the crystal structures currently available [18]). To determine which regions of UvrD were able to interact with UvrB we constructed plasmids that encoded the RNA polymerase α subunit fused to domains 1a and 1b, 2a and 2b, 1b alone, 2b alone, and the C-terminal extension of UvrD (Fig. 1A and Table 1). UvrB consists of five domains (Fig. 1B) [19,20]. Domains 1a and 3 are RecA-like domains that comprise the ATPase module of the protein. Domain 1a also participates in DNA damage-recognition, along with 1b [21]. Domains 2 and 4 are known to be involved in protein–protein interactions during NER: domain 2 interacts with UvrA [22], and domain 4 interacts with both UvrA and UvrC [23]. To determine which regions of UvrB were able to interact with UvrD we constructed plasmids that encoded the λcI protein fused to a range of truncated UvrB derivatives (Fig. 1B and Table 1).

The reporter strain KS1 was transformed with combinations of *rpoA-uvrD* and *λcI-uvrB* expression plasmids, and β-galactosidase activity was measured (Table 1). The results suggest that two regions of UvrD interacted with UvrB in this assay. The N-terminal fragment of UvrD, which contained domains 1a and 1b, interacted with all three UvrB fragments tested, with the strongest interaction being made with UvrB domains 1b and 3. The C-terminal extension of UvrD (residues 645–720) interacted with the UvrB fragment containing domains 1a and 2, but did not interact with either of the other UvrB fragments.

**Fig. 2:**
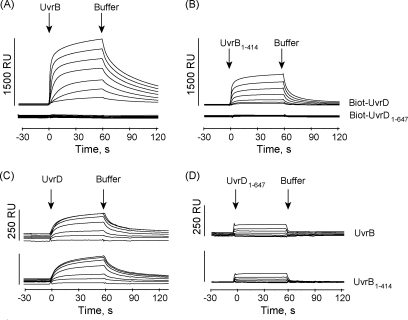
Analysis of the UvrD:UvrB interaction using surface plasmon resonance spectroscopy. (A) and (B): Biotinylated UvrD and UvrD_1–647_ were immobilised on the surface of a streptavidin-coated sensor chip and a range of concentrations of UvrB (A) or UvrB_1–414_ (B) in SPR buffer were passed over the chip surface. (C) and (D): UvrB and UvrB_1–414_ were covalently immobilized onto the CM5 sensor chip by amine coupling and a range of concentrations of UvrD (C) or UvrD_1–647_ (D) were passed over the chip surface. The concentrations of analyte used in all experiments were 0.05, 0.1, 0.2, 0.5, 1.0, 2.0 and 4.0 μM.

In parallel to the 2-hybrid approach, we analysed the interaction between UvrD and UvrB *in vitro* using surface plasmon resonance (SPR) spectroscopy. In order to be able to immobilise UvrD on a surface coated with streptavidin we constructed an expression vector that encodes UvrD with a 20 residue N-terminal tag. Co-expression of the BirA enzyme results in biotinylation of this tag *in vivo*, allowing the protein to be purified by affinity chromatography [8]. The purified biotinylated UvrD was immobilised on a streptavidin-coated chip, and then buffer containing UvrB at a range of concentrations was allowed to flow over the UvrD-modified surface. We observed a change in the SPR signal that increased with UvrB concentration, which indicates that UvrB bound to UvrD in this assay (Fig. 2A). A truncated derivative of UvrB comprising domains 1a, 2 and 1b (UvrB_1–414_) also bound to UvrD (Fig. 2B). Additional SPR experiments were conducted in the reverse orientation: amine coupling was used to immobilise UvrB or UvrB_1–414_ on the surface of a carboxymethylated dextran (CM5) chip, and then buffer containing non-biotinylated UvrD was allowed to flow over the UvrB-modified surface. We observed a change in the SPR signal that increased with UvrD concentration, which indicates that UvrD bound to both UvrB and UvrB_1–414_ in this assay (Fig. 2C). These results confirm the previous finding that UvrB and UvrD can bind to one another *in vitro* [6], and, in agreement with the results of our bacterial 2-hybrid assay, indicate that the N-terminal region of UvrB comprised of domains 1a, 2 and 1b contains a contact site(s) for UvrD.

The bacterial 2-hybrid screen had indicated that the C-terminal region of UvrD interacts with UvrB. To test this hypothesis we repeated the SPR experiments with a truncated version of UvrD that lacks the C-terminal 73 residues (UvrD_1–647_). Neither full-length UvrB nor UvrB_1–414_ generated a change in SPR signal when added to a streptavidin chip coated with biotinylated UvrD_1–647_ (Fig. 2A and B), indicating that these proteins cannot bind to UvrD that lacks the C-terminal region. When the experiments were conducted in the reverse orientation (i.e. UvrB or UvrB_1–414_ were immobilised on a CM5 chip and non-biotinylated UvrD_1–647_ was allowed to flow over the surface) a small change in the SPR signal, possibly indicative of interaction, was detected (Fig. 2D). These results might reflect the different restrictions that the two types of chip place on protein orientation and the fact that both the C-terminal region and domain 1a of UvrD are expected to contribute to interactions between UvrB and UvrD in these experiments. When UvrB was immobilised on the CM5 chip (Fig. 2C, D) it will have been present on the surface in multiple orientations, subsets of which may have interacted with the UvrD molecules from solution via domain 1a, the C-terminal region, or both. When biotinylated UvrD was immobilised on the chip surface (Fig. 2A, B) all of the molecules were present in a single orientation, and were tethered to the surface via a tag attached to domain 1a. It is possible that this obscured the UvrB-binding site in domain 1a and so rendered the interaction of the two proteins on the streptavidin chip completely dependent on the C-terminal region of UvrD.

### 3.2 Ability of UvrD_1–647_ to function in NER

During NER UvrD displaces both UvrC and the short excised oligonucleotide that contains the DNA damage from the UvrB:UvrC:DNA post-incision complex [24–27]. The experiments described above indicate that the C-terminal region of UvrD interacts with the N-terminal region of UvrB. To determine whether this interaction is important for the function of the proteins during NER we examined the ability of the purified UvrD_1–647_ to catalyse these reactions *in vitro*.

**Fig. 3:**
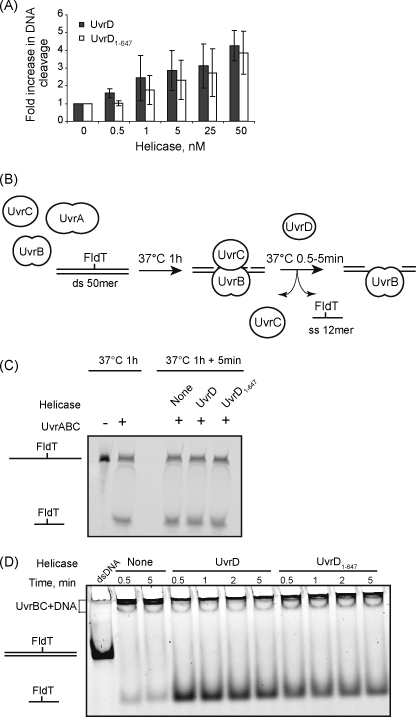
Analysis of the ability of UvrD_1–647_ to promote UvrC-turnover and to displace the excised oligonucleotide from a post-incision complex. (A) UvrC-turnover experiments were performed using 1.2 nM UV-irradiated plasmid DNA, 6 nM UvrA, 30 nM UvrB, 3 nM UvrC and indicated concentrations of UvrD and UvrD_1–647_. The amount of plasmid converted from supercoiled to nicked form after 10 min incubation was measured, and is expressed relative to the amount of DNA nicked in the absence of helicase. Values shown are the average of 3 independent experiments, with standard deviation. (B) A schematic representation of the assay used to monitor the displacement of the FldT-containing oligonucleotide from a post-incision complex. 10 nM FldT-containing 50mer was incubated with 40 nM UvrA, 200 nM UvrB and 100 nM UvrC for an hour at 37 °C, then the samples were split and buffer, 10 nM UvrD or 10 nM UvrD_1–647_ was added. Samples were taken at intervals for analysis by denaturing and native PAGE. (C) Analysis of DNA cleavage in post-incision complexes incubated with or without helicases. Samples were analysed on a 10% polyacrylamide/7 M urea gel and fluorescently labelled DNA was detected using a Molecular Dynamics Typhoon imager. (D) Displacement of the excised oligonucleotide from a post-incision complex in the presence or absence of helicases. Samples were analysed on a 10% native polyacrylamide gel containing 10 mM MgCl_2_, and fluorescently labelled DNA was detected as above. The gels shown are representative of at least 3 independent experiments.

The UvrC-displacement activity of UvrD and UvrD_1–647_ was assayed by monitoring cleavage of UV-irradiated plasmid DNA by UvrA, B and C, under conditions where UvrC concentration was limiting. Previous work has shown that UvrD increases the proportion of DNA molecules that are cleaved under such conditions, because it causes turnover of UvrC molecules that would otherwise be sequestered in post-incision complexes [24–27]. We added a range of concentrations of UvrD or UvrD_1–647_ to reactions in which the concentration of UvrC was insufficient to allow cleavage of all of the plasmid molecules present (Fig. 3A). In accordance with the previous work we found that addition of full-length UvrD increased the proportion of plasmid molecules that had been cleaved by the NER proteins after 10 min incubation. UvrD_1–647_ also increased the amount of UV-irradiated DNA cleaved by the NER proteins when UvrC concentration was limiting, which indicates that the interaction between UvrB and the C-terminal region of UvrD is dispensable for the ability of UvrD to displace UvrC from the post-incision complex.

To examine the ability of UvrD to displace the damage-containing oligonucleotide from a post-incision complex we used a 50mer duplex substrate carrying a single flourescein-dT (FldT) adduct close to the centre of one strand. In this assay the FldT adduct had two functions: it was recognised as a DNA lesion by the bacterial NER apparatus, and it was the label that allowed the oligonucleotides to be detected. The FldT-containing substrate was incubated with UvrA, UvrB and UvrC for 1 h to allow DNA damage-recognition and incision to proceed to completion. The post-incision complexes were then incubated for a further 30 s to 5 min with or without UvrD (or UvrD_1–647_) to allow oligonucleotide displacement to occur (Fig. 3B). Aliquots from each sample were analysed separately for cleavage and for oligonucleotide displacement.

To determine what proportion of the DNA was cleaved, the samples were analysed by denaturing gel electrophoresis (Fig. 3C). In each case approximately half of the 50mer substrate had been cleaved by UvrC to release a 12mer product, and the efficiency of cleavage was unaffected by the presence of UvrD or UvrD_1–647_ (as expected, because UvrC was present in excess in these reactions). To monitor the release of the excised oligonucleotide from the post-incision complex samples were analysed by non-denaturing PAGE (Fig. 3D). In the absence of UvrD or UvrD_1–647_ the majority of the fluorescent label remained close to the well (presumably as part of the UvrB:UvrC:DNA complex), and little free 12mer oligonucleotide was detected. When UvrD was added to the post-incision complexes the 12mer containing the FldT adduct was released from the complex and ran on the gel as free oligonucleotide. Under the conditions of our assay this reaction was complete within 30 s. UvrD_1–647_ also rapidly released the adduct-containing oligonucleotide from the post-incision complex, although with slightly reduced efficiency. These results are consistent with previous reports that UvrD is required to displace the oligonucleotide from the post-incision complex [24–26], and indicate that the C-terminal region of UvrD is dispensable for this activity.

**Fig. 4:**
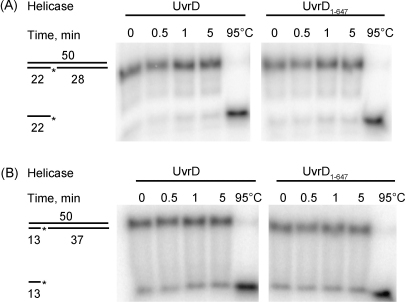
Helicase activity of UvrD and UvrD_1–647_ under the conditions used to monitor displacement of the FldT-containing oligonucleotide from post-incision complex assays. The reactions were started by adding 10 nM helicase to a reaction mix containing 10 nM nicked oligonucleotide substrates. Samples were removed at intervals, terminated with stop buffer, and analysed on 20% native polyacrylamide gels. (A) Substrate: 5′ γ-^32^P-labelled 22mer and unlabelled 28mer annealed to 50mer. (B) Substrate: 5′ γ-^32^P-labelled 13mer and unlabelled 37mer annealed to 50mer. The ^32^P-label is indicated by an asterisk. The gels shown are representative of at least 3 independent experiments.

To determine whether the ability of UvrD to displace short oligonucleotides from nicked DNA templates was facilitated by the other components of the post-incision complex (i.e. UvrB and/or UvrC) we examined the ability of UvrD to displace a 22mer or a 13mer from a nicked double-stranded duplex in the absence of other proteins, under the reaction conditions that had been used for the analysis of oligo-displacement from the post-incision complex (Fig. 4A and B). We observed little or no helicase activity from either UvrD or UvrD_1–647_ in these experiments: the amount of free oligonucleotide remained essentially unchanged even after 5 min incubation with the enzymes. Taken together our results indicate that UvrD is more efficient at displacing an oligonucleotide from a post-incision NER complex than it is at displacing a similar length oligonucleotide from a naked nicked template. This may indicate that interactions between UvrD and the other proteins in the post-incision complex facilitate recruitment or loading of UvrD, or stimulate its helicase activity. Alternatively it may indicate that the DNA in the post-incision complex adopts a conformation in which bending or partial unwinding of the duplex destabilises the interactions between the oligonucleotide and its complementary strand, or aids recruitment or loading of UvrD.

**Fig. 5:**
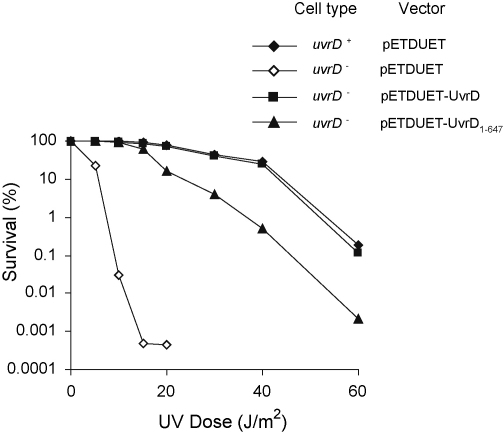
Effect of UvrD and UvrD_1–647_ on UV sensitivity of *uvrD*^*−*^ cells. Survival of *uvrD*^*+*^ (BW25113) or *uvrD*^*−*^ (JW3786-5) strains transformed with the indicated plasmids were determined after irradiation with the indicated doses of 254 nm UV light. Strains transformed with pETDUET are controls that do not express a plasmid-borne *uvrD* gene. Data points are the average of at least 3 experiments, each conducted in triplicate.

We tested the ability of UvrD_1–647_ to function in NER *in vivo* by monitoring its ability to restore UV-resistance to a strain that lacked chromosomal *uvrD*. The UvrD expression plasmids created in this work require T7 RNA polymerase for high level expression, but western blotting with a polyclonal antibody raised against UvrD confirmed that both UvrD and UvrD_1–647_ were expressed at a detectable level from these plasmids in strain JW3786-5 (*ΔuvrD769∷kan*), which lacks the T7 RNA polymerase gene (data not shown). We presume that this expression results either from low level transcription initiation by *E. coli* RNA polymerase at the T7 promoter region, or from transcription from cryptic promoters in the vector sequence upstream of the cloned *uvrD* gene. As expected [28], the *uvrD*^*−*^ strain was far more sensitive to UV-irradiation than a control strain that expressed UvrD from the chromosomal allele (Fig. 5). Expression of UvrD from pETDUET-UvrD restored the UV-resistance of the *uvrD*^*−*^ strain to the wild-type level. Expression of UvrD_1–647_ from pETDUET-UvrD_1–647_ substantially increased the UV-resistance of the *uvrD*^*−*^ strain, but did not completely restore the wild-type phenotype. These findings show that the C-terminal region of UvrD is not essential for the activity of the protein during NER *in vivo*, but that its contribution to UvrD:UvrB interactions may be physiologically relevant, particularly at high UV doses.

### 3.3 Biochemical characterisation of UvrD_1–647_

Deletion of 40 aa from the C-terminus of *E. coli* UvrD does not disrupt the helicase activity of the protein [29], but such a truncation does not remove the entire unstructured region. We therefore further examined UvrD_1–647_ to determine how removal of the entire unstructured C-terminal region of UvrD affected the properties of the protein.

The DNA-dependent ATPase activity of UvrD_1–647_ was determined using an enzyme-linked assay at a range of concentrations of single-stranded poly(dT) DNA (Table 2). The apparent *k*_cat_ for ATP hydrolysis was slightly higher than that measured for the wild-type enzyme, but the apparent affinity of UvrD_1–647_ for ssDNA (judged by the concentration of poly(dT) required for half-maximal ATPase activity) was more than 4-fold higher than wild-type. Deletion of the C-terminal region of UvrD thus appears to reduce the affinity of the protein for ssDNA, but does not substantially alter the ATPase activity of the protein.

**Fig. 6:**
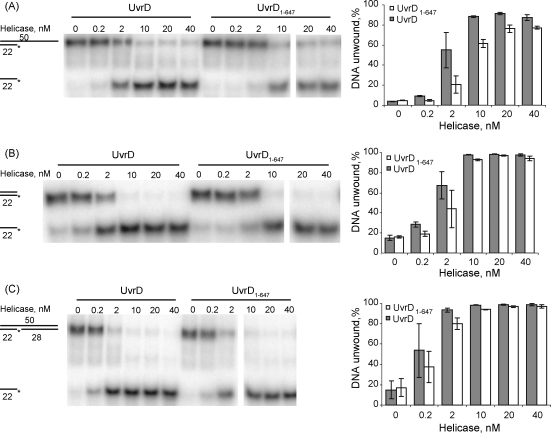
Helicase activity of UvrD and UvrD_1–647_ on various substrates under “low salt” conditions (50 mM KCl). The reactions were started by adding ATP to mixtures containing 2 nM labelled DNA substrate and the indicated concentrations of UvrD and UvrD_1–647_. Reactions were incubated for 10 min at 37 °C then terminated and analysed as in Fig. 4. The substrates analysed were: (A) a 3′ single-strand tailed substrate; (B) a blunt-ended 22 bp duplex; (C) a blunt-ended nicked 50 bp duplex. Each substrate contained a 5′ γ-^32^P-labelled 22mer, and the label is indicated by an asterisk. The data in the histograms are the average of 3 independent experiments, with standard deviation.

Under the conditions used for the analysis of oligo displacement from the post-incision NER complex neither full-length UvrD nor UvrD_1–647_ exhibited helicase activity on a naked nicked substrate. To examine the helicase activity of the proteins in the absence of the other Uvr proteins we conducted oligo-displacement assays at a lower salt concentration (50 mM KCl rather than 100 mM KCl). The helicase activity of UvrD_1–647_ was tested on a range of substrates on which UvrD is expected to be active [5,30]: a 22 bp dsDNA region with a 38 nt ssDNA 3′ tail (Fig. 6A); a 22 bp blunt-ended duplex (Fig. 6B); and a 50 bp duplex containing a nick 22 bp from the 3′ end of one strand (Fig. 6C). In each case UvrD_1–647_ was able to unwind the substrates, although a slightly higher concentration of UvrD_1–647_ than wild-type UvrD was required to unwind the 3′ tailed template, which is consistent with the reduced affinity of the truncated protein for single-stranded DNA that was observed during the ATPase assays.

### 3.4 Ability of other superfamily 1A helicases to substitute for UvrD in NER

The *E. coli* Rep protein shares approximately 40% sequence identity with UvrD, and the two proteins have overlapping activities: strains lacking either *rep* or *uvrD* are viable, but deletion of the two genes simultaneously is lethal [31]. However, Rep is not able to substitute for UvrD in NER: strains that lack UvrD are UV-sensitive, and purified Rep does not displace UvrC from post-incision complexes [26]. Gram-positive organisms such as *Bacillus subtilis* and *Bacillus stearothermophilus* contain an essential Rep/UvrD-like helicase called PcrA, which is approximately 40% identical to both Rep and UvrD from *E. coli* at the primary sequence level [32,33]. Expression of *B. subtilis* PcrA in *E. coli* restored the UV-resistance of a *uvrD* strain [32], which suggests that PcrA can function during NER and can cooperate with *E. coli* NER proteins, but this has not been tested biochemically.

**Fig. 7:**
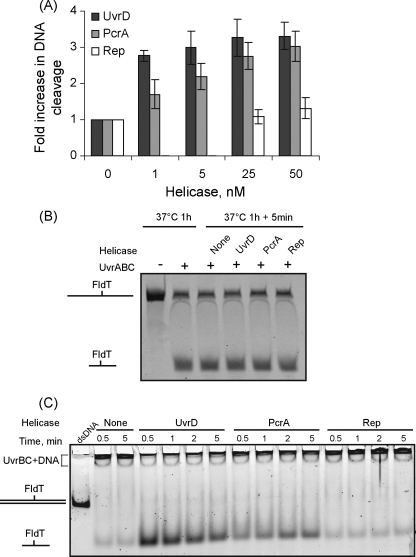
Effects of PcrA and Rep on post-incision NER complexes. (A) UvrC-turnover experiments were performed using 1.2 nM UV-irradiated plasmid DNA, 6 nM UvrA, 30 nM UvrB, 3 nM UvrC and indicated concentrations of helicases in repair buffer. Values shown are the average of 3 independent experiments, with standard deviation. (B) Analysis of DNA cleavage in post-incision complexes incubated with or without helicases. Samples were analysed as in Fig. 3C. (C) Displacement of the excised oligonucleotide from a post-incision complex in the presence or absence of helicases. Samples were analysed as in Fig. 3D. The gels shown are representative of at least 3 independent experiments.

To gain further insight into the specificity of helicase action during NER we examined the ability of *B. stearothermophilus* PcrA and *E. coli* Rep to displace UvrC and the damage-containing oligonucleotide from post-incision complexes using the *in vitro* assays that have been described above. We found that PcrA was able to catalyse UvrC-turnover, as judged by nicking of UV-irradiated DNA under UvrC-limiting conditions (Fig. 7A). PcrA was also able to displace the damaged oligonucleotide from the post-incision complex, albeit rather less efficiently than UvrD (Fig. 7B and C). In contrast, Rep did not displace the damaged oligonucleotide from the post-incision complex (Fig. 7C) and, as shown previously [26], it did not stimulate UvrC-turnover (Fig. 7A). Control experiments confirmed that the preparations of PcrA and Rep used in these experiments exhibited helicase activity on a 3′-tailed substrate (Supplementary Fig. 1).

## 4 Discussion

The interaction between UvrB and UvrD has been demonstrated previously by co-immunoprecipitation, and has been suggested to recruit UvrD to the post-excision NER complex [6]. In this work we have confirmed the interaction of UvrB with UvrD by 2-hybrid analysis *in vivo* and by SPR spectroscopy *in vitro*. Our results suggest that two regions of UvrD contribute to the interaction with UvrB, and that the contact patch for UvrD lies, at least in part, in domain 1a, 2 or 1b of UvrB. One region of UvrD that interacts with UvrB is domain 1a, which is the N-terminal RecA-like domain that forms half of the UvrD ATPase module (a fragment of UvrD comprising domains 1a and 1b interacted with all of the subfragments of UvrB tested in the 2-hybrid assay, but the isolated 1b domain did not). The other region of UvrD that interacts with UvrB is the unstructured C-terminal extension. This pattern of interactions is reminiscent of that observed in the interaction of UvrD with the MMR protein MutL [4]. Deletion of 100 amino acids from the N-terminus of UvrD or 40 amino acids from its C-terminus abolished the interaction with MutL in a yeast 2-hybrid assay. The helicase activity of UvrD is stimulated by both MutL (reviewed in [3]) and by UvrA/UvrB [5], and the apparent similarity between the UvrB:UvrD interaction and the MutL:UvrD interaction may indicate that a similar stimulatory mechanism functions in the two different situations.

Domain 1a is essential for the ATPase and helicase activity of UvrD, and so analysis of its interaction with UvrB will require the isolation of single-amino acid substitutions that specifically disrupt the interaction. The C-terminal extension of *E. coli* UvrD is one of the features that distinguishes UvrD (which functions in NER) from Rep (which does not), and it can be readily deleted from the protein. We therefore chose to focus our study on this region. In this work we have analysed the properties of UvrD_1–647_, in which the entire C-terminal extension was removed but domain 2a was not disrupted. UvrD_1–647_ is an active ATPase and retains helicase activity on a diverse range of DNA substrates. The truncated protein is also able to displace UvrC and the damage-containing oligonucleotide from the post-excision complex. We conclude that neither the C-terminal extension, nor its interaction with UvrB, are essential features of the mechanisms by which UvrD catalyses these reactions. The simplest interpretation of our results is that the interaction between UvrB and the C-terminal region of UvrD contributes to the affinity of the two proteins for one another, but that in the absence of this interaction the remaining contacts between UvrB and the N-terminus of UvrD are sufficient to support UvrD function in NER both *in vitro* and *in vivo*. This situation provides parallels with a recent study of Mycobacterial UvrD1, whose helicase activity is stimulated by interaction with the DNA end-binding protein, Ku [34]. Experiments with a truncated UvrD1 derivative that lacked the 90aa C-terminal extension (a construct equivalent to residues 1–650 of *E. coli* UvrD) showed that the C-terminus of the protein contributed to its interaction with Ku, but was not essential for Ku-UvrD1 interactions as the truncated protein was still stimulated by Ku in helicase assays [35].

The absence of the C-terminal extension in UvrD_1–647_ reduced the apparent affinity of the protein for single-stranded DNA. The effect of deleting either 40 or 102 residues from the C-terminus of *E. coli* UvrD (creating UvrDΔ40C and UvrDΔ102C) has been examined previously, and that work also implicated the C-terminal region of the protein in binding single-stranded DNA [29,36]. UvrDΔ40C and UvrDΔ102C both showed weakened binding to single-stranded DNA-cellulose columns, but in filter binding assays only UvrDΔ102C showed reduced affinity for single-stranded DNA [29]. UvrDΔ40C, lost the ability to dimerise, but was otherwise functional: it retained helicase activity and the ability to restore the UV-resistance of a strain lacking *uvrD*. In contrast UvrDΔ102C lacked ATPase activity as well as DNA-binding ability [29]. When the structure of UvrD was determined it became clear that deletion of 102 C-terminal residues not only removes the entire C-terminal extension, it also deletes part of domain 2a, including a conserved motif that is involved in DNA binding [18]. The loss of ATPase and DNA binding activity therefore seemed likely to be due to disruption of domain 2a, rather than loss of the C-terminal extension. Our observation that UvrD_1–647_, which retains domain 2a, shows reduced affinity for single-stranded DNA provides fresh evidence for a role of the C-terminal region of UvrD in single-stranded DNA binding.

One of the motivations for undertaking this study was to try to understand the specificity of helicases for particular roles in the cell. UvrD and Rep are similar proteins and are partially redundant [31], but Rep cannot substitute for UvrD in NER *in vivo*, and in this work we have shown that Rep cannot displace the damage-containing oligonucleotide from a post-incision NER complex *in vitro*. It has been suggested that in Gram-positive organisms the roles of both UvrD and Rep are combined in a single essential helicase, PcrA [32], and we have shown that PcrA can displace both UvrC and the damage-containing oligonucleotide from a post-incision NER complex. What is the basis for this specificity? The finding that the C-terminal region of UvrD interacts with UvrB may provide part of the answer, as Rep does not contain an equivalent C-terminal region, and PcrA does. However the ability of UvrD_1–647_ to function in NER indicates that the critical distinguishing factor between the proteins remains to be discovered.

## Conflict of interest statement

The authors declare that there are no conflicts of interest.

## Figures and Tables

**Table 1:** Analysis of the UvrD:UvrB interaction using a bacterial 2-hybrid assay. *E. coli* KS1 was transformed with the indicated combinations of plasmids pRA02 and pRA03, or derivatives. Row headings indicate domains of UvrD fused to a truncated RNA polymerase α subunit, expressed from pRA02 derivatives (pRA02 encodes the α construct without additional fused domains). Column headings indicate domains of UvrB fused to the λ cI protein, expressed from pRA03 derivatives (pRA03 encodes the λ cI construct without additional fused domains). Values shown are specific β-galactosidase activities determined during mid-log growth of the transformed strains, and are expressed as Miller Units [13]. Values shown in roman text are the averages of at least 3 independent experiments, with standard deviation. Values shown in italic text are the range obtained from 2 independent experiments.

UvrD	UvrB			
	35–252 (1a2)	251–547 (1b3)	527–673 (4)	pRA03 (no UvrB)
1–280 (1a1b1a)	111 ± 3	336 ± 62	86 ± 12	32 ± 7
83–214 (1b)	31 ± 7	*31–30*	*17–20*	*16–18*
280–645 (2a2b2a)	32 ± 3	*45–45*	*38–44*	*29–35*
377–550 (2b)	32 ± 5	*33–38*	*18–26*	*23–27*
645–720 (C-extension)	140 ± 16	42 ± 5	24 ± 2	33 ± 9
pRA02 (no UvrD)	31 ± 4	46 ± 4	23 ± 3	37 ± 2

**Table 2:** DNA-dependent ATPase activity of UvrD_1–647_. The ATPase activity of UvrD and UvrD_1–647_ were measured at a range of poly(dT) concentrations from 0 to 16 μM nucleotides. The data were plotted and used to calculate an apparent *k*_cat_ for ATP hydrolysis and a *K*_DNA_ (concentration of poly(dT) required for half-maximal ATPase rate) for the interaction of the proteins with DNA. The values shown are the averages of 3 independent experiments, with standard error.

Helicase	*k*_cat_ (s^−1^)	*K*_DNA_ (μM nucleotides)
UvrD	389.8 ± 6.3	0.54 ± 0.03
UvrD_1–647_	497.5 ± 7.9	2.5 ± 0.1
