# Supplementary material for: The unstructured C-terminal extension of UvrD interacts with UvrB, but is dispensable for nucleotide excision repair
Source: DNA Repair (Amst). 2009 Nov 2;8(11):1300–10. doi: 10.1016/j.dnarep.2009.08.005 (PMC2997466; doi:10.1016/j.dnarep.2009.08.005)
Supplement: Supplementary file 1 [file mmc1.pdf]

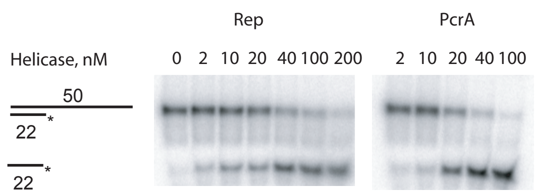

Manelyte et al. Supplementary Figure 1

Helicase activity of Rep and PcrA on a 3′ single-strand tailed substrate under “low salt” conditions (50 mM KCl). The reactions were started by adding ATP to mixtures containing 2 nM labelled DNA substrate and the indicated concentrations of Rep and PcrA . Reactions were incubated for 10 min at 37°C then terminated and analysed as in Figure 4. The substrate contained a 5’ γ-32P-labelled 22mer, and the label is indicated by an asterix.
